# miRNAs as Biomarkers in Disease: Latest Findings Regarding Their Role in Diagnosis and Prognosis

**DOI:** 10.3390/cells9020276

**Published:** 2020-01-23

**Authors:** Carmen Elena Condrat, Dana Claudia Thompson, Madalina Gabriela Barbu, Oana Larisa Bugnar, Andreea Boboc, Dragos Cretoiu, Nicolae Suciu, Sanda Maria Cretoiu, Silviu Cristian Voinea

**Affiliations:** 1 Alessandrescu-Rusescu National Institute for Mother and Child Health, Fetal Medicine Excellence Research Center, 020395 Bucharest, Romania; drcarmencondrat@gmail.com (C.E.C.); dana.lunganu@gmail.com (D.C.T.); mada.barbu93@gmail.com (M.G.B.); oanabugnar@yahoo.com (O.L.B.); boboc_elena_andreea@yahoo.com (A.B.); dragos@cretoiu.ro (D.C.); nsuciu54@yahoo.com (N.S.); 2 Department of Cell and Molecular Biology and Histology, Carol Davila University of Medicine and Pharmacy, 8 Eroii Sanitari Blvd., 050474 Bucharest, Romania; 3 Division of Obstetrics, Gynecology and Neonatology, Carol Davila University of Medicine and Pharmacy, 8 Eroii Sanitari Blvd., 050474 Bucharest, Romania; 4 Department of Obstetrics and Gynecology, Polizu Clinical Hospital, Alessandrescu-Rusescu National Institute for Mother and Child Health, 020395 Bucharest, Romania; 5 Department of Surgical Oncology, Prof. Dr. Alexandru Trestioreanu Oncology Institute, Carol Davila University of Medicine and Pharmacy, 252 Fundeni Rd., 022328 Bucharest, Romania; dr.voineasilviu@gmail.com

**Keywords:** miRNA, biomarker, diagnosis, prognosis, cancer, cardiovascular disease, sepsis, nervous system

## Abstract

MicroRNAs (miRNAs) represent a class of small, non-coding RNAs with the main roles of regulating mRNA through its degradation and adjusting protein levels. In recent years, extraordinary progress has been made in terms of identifying the origin and exact functions of miRNA, focusing on their potential use in both the research and the clinical field. This review aims at improving the current understanding of these molecules and their applicability in the medical field. A thorough analysis of the literature consulting resources available in online databases such as NCBI, PubMed, Medline, ScienceDirect, and UpToDate was performed. There is promising evidence that in spite of the lack of standardized protocols regarding the use of miRNAs in current clinical practice, they constitute a reliable tool for future use. These molecules meet most of the required criteria for being an ideal biomarker, such as accessibility, high specificity, and sensitivity. Despite present limitations, miRNAs as biomarkers for various conditions remain an impressive research field. As current techniques evolve, we anticipate that miRNAs will become a routine approach in the development of personalized patient profiles, thus permitting more specific therapeutic interventions.

## 1. Introduction

microRNAs (miRNAs) represent a class of small, non-coding RNAs comprising of 17–25 nucleotides [1], whose main role is to regulate mRNA by leading to its degradation and also to adjust the protein levels [1,2,3,4]. Their discovery was first published in 1993 and they were described as “mediators of temporal pattern formation” in *Caenorhabditis elegans* [5,6,7,8,9]. Previous studies have shown that miRNA encoding sequences form up to 1% of the human genome [10].

Biogenesis of miRNA begins in the nucleus, where the transcription of its precursor, primary miRNA or pri-miRNA takes place under the influence of RNA polymerases II and III [11,12]. The resulting molecule is a hairpin-like structure, which contains a loop at one end [11]. This primordial mi-RNA precursor that is usually made up of hundreds of nucleotides is then processed consecutively by two RNase III enzymes [13,14,15]. The first enzyme to act upon the pri-miRNA, which still resides in the nucleus, is called Drosha or DCGR8, and turns it into a new hairpin-like structure of approximately 70 nucleotides, the Precursor-miRNA or pre-miRNA. The latter is then transported to the cytoplasm, with the help of Exportin-5, where it is cleaved again by the Ago2/Dicer complex leading to the short, mature miRNA double strands [16].

Further on, one of the strands, usually known as the guide strand, will be integrated into the RNA-induced silencing complex (RISC), while the other one, known as the passenger strand, is going to be degraded, even though in some occasions it has been found to be also functional [17]. In most cases, the strand that contains the less stable 5′ end or a uracil at the beginning is more likely to be selected as the guide strand [18,19,20]. In those situations, where the passenger strand is not degraded and both get incorporated into the miRISC complex, the mature miRNA in the guide strand will be the dominant one [21,22].

The main role of miRNA in the human body is gene regulation [23] by mediating the degradation of mRNA and also by regulating transcription and translation through canonical and non-canonical mechanisms [4]. The canonical mechanism means that the miRISC complex containing the miRNA guide strand is exerting its action by binding to the target mRNA through its 3′-untranslated region (3′-UTR) [3]. This process happens in accordance with the seed sequence of the miRNA, the first 2-7 nucleotides from the 5′ end, and it is followed by mRNA deadenylation, translation suppression and finally, degradation [24,25,26]. However, in human cells, about 60% of the interactions between the miRISC complex and mRNA are non-canonical [27], which means that their chains are not always entirely complementary [28]. This leads to the idea that a single miRNA could potentially target numerous mRNAs, while at the same time, one mRNA could contain multiple binding sites for miRNAs, turning this into a possibility that vast number of biological processes could be regulated by this interaction [3].

Another important role played by miRNA is intercellular signaling. Even though most of the miRNAs are found inside the cell, there is a big proportion that migrates outside it and can be found in bodily fluids [29,30,31,32,33]. These are the so-called circulating miRNAs and they are discharged in blood, urine, saliva, seminal fluid, breast milk [30,34] and other fluids through tissue damage, apoptosis, and necrosis [4], or through active passage, in microvesicles, exosomes, or through bonding to a protein [35,36]. The question has also been raised regarding the existence of exogenous miRNA in the blood of healthy subjects [37,38], its origin being assigned to bacteria, food and fungi primarily from the gut [3]. The possible pathological effects of these exogenous miRNA are also taken into consideration.

Previous studies have shown that about 10% of the circulating miRNAs are secreted in exosomes, while the other 90% form complexes with proteins like argonaute 2 (Ago2), nucleophosmin 1 (NPM 1) and high density lipoprotein (HDL) [36,39,40]. This kind of packaging is essential in order to prevent the digestion of miRNA, by the RNases known to be found in the bodily fluids [41].

Having a diameter of 50–100 nm, exosomes are a form endosome-derived microvesicles fusing with the membrane, which contains, amongst others, miRNA. They are secreted by numerous cells, both in vitro and in vivo and can be encountered in most of the fluids in the body [30,34]. Several previous studies have demonstrated in vitro the assimilation of exosomes by neighboring cells, thus leading to the idea of a “message in a bottle” type intercellular communication [42,43,44]. Furthermore, another hypothesis supported by recent findings states that miRNAs could be selectively designated to certain exosomes because of the fact that the miRNA variety found in these vesicles tends to be different from that of their cell of origin [42,45,46,47].

As mentioned earlier, the transport with the help of a protein of circulating miRNAs is estimated at around 90% of the total. Some papers suggest that this type of transport could be ATP-dependent [36], while others indicate that in order for the complex to reach the extracellular space, it requires a transporter, similar to the well-known protein-specific mechanism [31]. Both these hypotheses need more research before being proven correct.

In recent years, extraordinary progress has been made in terms of finding the origin and functions of miRNA and their potential use in research and clinical practice, for both healthy and diseased patients. For example, some studies have shown that the levels of certain circulating miRNAs, involved in inflammation, angiogenesis, and cardiac muscle contractility, are directly influenced by the intensity and length of exercising, thus indicating that they might play a role in mediating the physiological cardiac adaptation to exercising [48,49,50]. Another study has shown the key role miRNAs are playing in the creation of induced pluripotent stem cells (iPS). In this case, miRNAs are repressed by reprogramming factors, turning average fibroblasts into iPS cells [51].

However, probably the most promising role of miRNAs is that of a potential biomarker. Numerous authors have investigated this opportunity in various medical fields. Evidence suggests that they could play an essential role as biomarkers in cancer through exosome-mediated intercellular communication [1,4,52,53,54], in neurology for the diagnosis and prognosis of Alzheimer’s disease [55], for patients with spinal cord injury [56], epilepsy [57] or neurodegenerative ailments [58]. It could also be used in other fields like cardiology, as a faster and more accurate means of diagnosis for acute cardiovascular disease or heart failure [59,60,61] and in the case of infectious diseases for the diagnosis of sepsis [62]. Although current literature on miRNAs does not seem to lack in writings emphasizing the numerous roles of these molecules both in general [63,64,65] and in specific areas such as oncology [66,67,68], cardiovascular diseases [69,70], sepsis [71], our aim is to offer an overview on the current state of the knowledge while occasionally mentioning areas of controversy.

## 2. The Potential of miRNAs as Biomarkers

Biomarker is a term that defines different types of objective indicators of health or disease. Throughout history, and according to human technological advancements, these indicators have turned increasingly more precise and reliable. Some of the first biomarkers were discovered in ancient times and were represented by the medical signs, such as pulse, the looks and even the taste of urine.

Nowadays, the medical community refers to biomarkers as being certain molecules, usually proteins, detected in the various fluids of the human body, through specific means in medical laboratories. The best known protein biomarkers measurable in the blood are troponin for the diagnosis of myocardial infarction, carcinoembryonic antigen (CAE) for different types of cancer, aminotransferases ALT and AST for liver diseases and the prostate-specific antigen (PSA) for the diagnosis and prognosis of prostate cancer [2]. Recently, however, detecting new and improved protein biomarkers has turned out to be a time consuming and expensive operation, due to the low amount of clinically significant proteins, the complexity of their structure and the struggle in finding accurate detection methods.

In order to attain the desiderates of personalized medicine, new and more accurate biomarkers need to be discovered. An ideal biomarker needs to fit certain criteria. First of all, it needs to be easily accessible, which means that it needs to be discovered and measured through minimally invasive procedures. Another important criterion is the specificity to the investigated pathology, followed by sensitivity (its presence should be detected preferably before the clinical symptoms have appeared and should vary according to the disease progression or response to treatment). Last, but not least, it should be translatable from research to clinic [72].

Researchers have discovered the existence of free nucleic acids in the blood for about 60 years [73,74,75,76,77], while DNA and RNA from tumors are frequently encountered in the plasma of cancer patients [78,79,80,81]. It was considered for a while that RNA molecules could not be used as biomarkers from blood samples, because of the elevated levels of nucleases found in plasma [82], but the idea was dismissed once it was discovered that miRNAs were stable in samples of fixed tissues [83].

miRNAs have first been established as biomarkers for cancer in 2008, when Lawrie et al. utilized them for the examination of diffuse large B-cell lymphoma in the serum of patients [84,85], and ever since, their potential use as biomarkers has been mentioned in literature for numerous diseases.

This novel class of molecules possesses an array of advantages that could turn them into ideal candidates for biomarkers in a variety of afflictions. As mentioned before, the ideal biomarker needs to be easily accessible, a condition that applies to miRNAs that can easily be extracted through liquid biopsies from blood, urine and other bodily fluids. It also has a high specificity for the tissue or cell type of provenance and it is sensitive in the way that it varies according to the disease progression, being used in several studies for the differentiation of the cancer stages [86] and even for the measurement of the therapy responsiveness [87]. Moreover, the technologies for the detection of nucleic acids already exist and the development of new assays requires less time and lower costs in comparison to producing new antibodies for protein biomarkers.

Another advantage of miRNAs lies in their potential for being used as multimarker models for accurate diagnosis, guided treatment and evaluating responsiveness to treatment. While running many protein markers may be both expensive and time-consuming, using multimarker panels composed of numerous miRNAs may provide a non-invasive method for diagnosis and prediction of disease progression. For instance, identifying the urinary miRNA signature of lupus nephritis has promoted the early detection of renal fibrosis [88]. This is especially important in cancer, a thoroughly heterogenous disease, where a multimarker approach would be preferable. To this extent, a nine-miRNA multimarker panel for breast carcinoma has already been shown to significantly improve the reliability of breast cancer diagnosis [89].

However, the research of miRNAs as biomarkers is still in its early stages, therefore at the moment, the findings generally lack reproducibility. There are several discordances reported between different teams that have analyzed the same tumors [90]. In order to resolve this issue, standardized protocols must be developed both for the initial stages of the process, like sample collection, transport, and storage, as well as data analyzing for the diversity of technological methods used.

## 3. miRNA Identification Techniques

In order to aid the diagnostic process and optimize treatment plans, research has delved into molecular testing, aiming to develop an efficient and cost-effective method for detecting miRNAs involved in some of the most common diseases worldwide, while also trying to identify new such molecules associated with these pathologies. Nevertheless, this quest has proven to be rather challenging, mainly due to the fact that the field of miRNAs itself is relatively new, and therefore traits such as detection limits, range of concentrations in body fluids, modulation depending on various parameters (age, gender, health/disease) are not yet clearly established [91].

The gold-standard for miRNA quantification is quantitative reverse transcriptase PCR (RT-qPCR) [91,92,93]. Stem-loop reverse transcription (RT)-based TaqMan microRNA assay is the main PCR technique used in research, with the advantage of having high sensitivity and specificity rates [94]. This is a two-step method, the first step requiring the binding of miRNA molecules by primers at the 3′ end in order to proceed to stem-loop reverse transcription [95]. For the second step of the technique, real-time PCR is used to quantify the miRNAs targeted [95]. Other available options are direct RT-based and poly (A) tailing-based SYBR miRNA assays [94]. The downside to these techniques is that sensing errors for the samples used can sometimes happen and also, during the amplification steps, there is high risk of contamination [91].

Northern blot hybridization represents another widely used alternative for quantitative assessments of miRNAs. This method involves the separation of the total amount of RNA on polyacrylamide gel that possesses the property of denaturation, followed by its transfer on a nylon membrane. After that, the RNA undergoes a process of UV cross-linking and, lastly, it is hybridized using a radioactive substance [96,97,98,99]. However, this technique tends to be strenuous, it necessitates large quantities of RNA and it has sometimes been reported to omit rare types of miRNA [99]. Under these circumstances, efforts have been made in order to improve the method, therefore leading to the possibility of using lower amounts of RNA and also shortening the execution time of the technique [100,101,102].

Two other methods are also in use for the same purpose of identifying the miRNAs. In situ hybridization or ISH is a technique that utilizes radioactive, fluorescent or dioxygenin probes to bind the desired RNA, therefore comparing the expression of miRNAs in various cells [103]. The disadvantages of ISH, however, are still significant and include laborious steps and long processes, with a predisposition towards errors [103]. The second method is next-generation sequencing or NGS. Despite the fact that this is a highly accurate technique that has the ability to detect single miRNAs with the precision of one nucleotide, its high costs lead to a limitation of this technique’s wider accessibility [99].

## 4. miRNAs in Cancer

Cancer is the most important cause of mortality worldwide. Even though detection in its early stages could lead to a better outcome for the survival of the patient, unfortunately, late detection still remains a major concern nowadays, leading to poor prognosis and a high mortality rate [104]. As a result, and in order to overcome this problem in the age of personalized medicine, new and more sensitive diagnosis methods, such as biomarkers, need to be developed.

Numerous reports have published throughout the years, hundreds of cases of deregulated miRNAs found in the plasma and serum of cancer patients, in comparison to healthy subjects [105] and a lot more studies have nominated circulating miRNAs as potential biomarkers for the diagnosis and prognosis of cancer [105,106,107,108]. The role that circulating miRNAs are playing in cancer is controlling the oncogenes (which is achieved through tumor suppressor miRNAs) and the tumor suppressors (through oncomiRs) [109]. Usually, the oncomiRs tend to be excessively represented (for example miR-17-92 or miR-21 clusters) [110,111], while the miRNAs with tumor suppression function (let-7 cluster), are expressed insufficiently [112]. These discoveries have been made experimentally, by inhibition or inducement of their function.

There are, however, miRNAs that can act both as suppressors or oncomiRs. For example, miR-155 was first considered an oncomiR for lymphoma and pancreatic cancer [113,114], but other evidence suggests that in the cases of ovarian and gastric cancers and melanoma, its expression is inhibited, therefore acting like a tumor suppressor [115,116,117].

There are various causes of the abnormal expression of circulating miRNAs in cancer. Around 50% of the miRNA coding genes are located in areas of the genome that are associated with cancer, which are translocated or amplified in malignancies [118]. Another reason is represented by the function variation of the enzymes involved in the biosynthesis of miRNA, like Drosha and Dicer 1 [119]. A decrease in the levels of these enzymes has been reported in the case of bladder [120] and ovarian cancers [121], while elevated levels are encountered in gastric [122] and cervical squamous cell neoplasms [123]. Lastly, the alteration of circulating miRNAs in cancer could also be caused by transcriptional errors of pri-miRNA [119].

Further on, this paper will describe several types of cancer and how their miRNA profiles could potentially lead to medical advancements in terms of an early diagnosis and a better profiling of these malignancies. A brief summary of some of the most important findings analyzed in this paper is provided in Table 1.

### 4.1. miRNAs in Breast Cancer

Breast cancer is the most frequent neoplasm affecting women and a major cause of mortality worldwide [146]. Depending on the existence and/or absence of various hormone receptors, such as estrogen receptor (ER), progesterone receptor (PR) and the receptor for the human epidermal growth factor (HER2), the disease can be classified in subtypes, the most aggressive one being triple-negative breast cancer (TNBC) [147]. Lately, efforts have been made in order to find miRNA biomarkers that could improve the diagnostic process, as well as the treatment. As it happens in all types of malignancies studied, miRNAs can have either tumorigenic or suppressing effects on the breast cancer cells [148]. Knowing which miRNAs are involved in breast malignancy and what role they play would not only help in diagnosing the disease and correctly stratifying the patients into risk groups, but it could also aid in the development of new therapeutic agents.

One of the most researched miRNAs in breast cancer is miR-10b, which in early studies was believed to be downregulated in malignant tissues compared to normal samples [124]. However, later research proved that it is in fact overexpressed, but only in metastatic disease [125]. It has been shown that its expression in non-metastatic disease induces cell invasion and metastasis, probably by increasing the expression of *RhoC* (Ras homolog gene family, member C), a known oncogene [125]. Furthermore, the quantity of *miR-10b* detected, positively correlated with the disease progression [125]. High levels of *miR-10b* were also found in ER-negative breast cancer, indicating a poor disease outcome [126].

A recent study offered the possibility of a new miRNA prognostic biomarker, namely *miRNA-196a* which was already known to be involved in numerous malignancies, among them being breast cancer [127,128]. Suggestive for its supposed oncogenic role, it has been shown that the polymorphism of a single particular nucleotide within its encoding gene (*MIR196A2*) leads to a decreased expression of *miR-196a* and a lower risk of breast cancer [129]. *miR-196a* was found to alter angiogenesis and apoptosis, two of the mechanisms involved in the formation of malignant tumors [130]. High levels of *miR-196a* were detected in drug-resistant ER-positive breast cancer and seemed to be a valid prognostic instrument for advanced and post-menopausal disease with estrogen receptors [127].

Another disease biomarker is *miRNA-4417* which was lately found to be a solid prognostic feature for triple-negative breast cancer (TNBC) [52]. TNBC has been defined by the absence of hormone receptors, thus making it unresponsive to current ER and HER2 targeting therapies [131]. As a result, the development of new potential therapies would have a great impact on the survival rate and could lead to a reduction in toxicity levels related to conventional chemotherapy. Located on chromosome 1p36, *miR-4417* was found to be downregulated in TNBC, simultaneously with the evolution of cells to a malignant stage, a fact associated with poor prognosis for the evolution of the disease [52]. Furthermore, when overexpressed in vitro, it was observed that *miR-4417* inhibited the migration and the tumorigenic roles of TNBC cells, raising the possibility of it being one of the future therapy agents that could be used in this type of cancer [52].

The association between miRNAs and drug-resistance was extensively researched. A recent study compared a number of 411 miRNAs expressed in 36 breast cancer cell lines in relation to 34 drugs, among which were Docetaxel, Paclitaxel, Tivantinib and Veliparib [149]. They found that *miR-187-5p* and *miR-106a-3p* were indicative of drug resistance to both Docetaxel and Paclitaxel and that *miR-556-5p* associated with sensitivity only to the latter [149]. Various other miRNAs were linked to drug-sensitivity, such as *let-7d-5p* and *miR-18a-5p* to Tivantinib, *let-7a-5p* to Bortezonib, *miR-135a-3p* to JNJ-707 and *miR-185-3p* to Panabinostat [149]. Other miRNAs found in relation to drug-resistance were *miR-637* to Tivantinib, *miR-182-5p* to Valiparib and *miR-629-5p* to Tipifarnib [149]. Therefore, miRNAs could be used in the future as markers for intrinsic drug resistance/sensitivity leading to a better understanding of the therapy needed for each particular case and as a result reducing overtreatment and therapy costs.

### 4.2. miRNAs in Ovarian Cancer

Ovarian cancer is one of the major malignancies in the gynecologic pathology, having high mortality rates worldwide, in part due to the fact that it has no specific clinical manifestation in the early stages, leading to late diagnosis and tumor metastasis [150,151,152]. In addition, it has increased recurrence and drug resistance rates, further affecting the 5-year survival, which was measured at 46.2% [153]. In consequence, finding new miRNA biomarkers that could be used for screening and early diagnosis of this disease might improve the outcome of the disease.

With this purpose, a case-control study on a small number of ovarian cancer patients was carried out, revealing 147 miRNAs with altered expression compared to case studies [154]. They detected an increased expression of *miR-30c1* and low expression levels of *miR-342-3p*, *miR-181a* and *miR-450b-5p* in the blood samples of ovarian cancer patients compared to controls [154]. Furthermore, after comparing their results to studies on other types of malignancies, they found that the expression models of miRNAs observed in the study differed from those obtained in a variety of other diseases, reaching the conclusion that the expression patterns were specific to ovarian cancer and were not influenced by chemotherapy or inflammation [154].

Other upregulated miRNAs in ovarian cancer tissues are *miR-199a*, *miR-200a*, *miR-200b*, *miR-200* [132] and *miR-200c*, the latter being highly expressed only in the serous epithelial type [133]. *miR-200* family was found to inhibit the transcriptional inhibitors of E-cadherin, ZEB1, and ZEB2, thus promoting E-cadherin expression and the transformation into epithelial cells of mesenchymal cells [134]. In addition, by decreasing the expression of *miR-200a*, ovarian cancer cells underwent epithelial-mesenchymal transition (EMT), leading to cell invasion and metastasis [134,135]. *miR-200* expression levels corroborated with disease recurrence and survival rates, predicting poor outcomes when low levels were detected [135]. Moreover, the *miR-200* cluster was also found to be involved in the inhibition of angiogenesis by targeting IL-8 and CXCL-1 produced by the tumor cells [134]. Providing proof of this, researchers transferred *miR-200* in tumor epithelium, resulting in a critical decrease in angiogenesis and tumor metastasis [155]. Also a modulator of E-cadherin, *miR-506* was proved to inhibit cell invasion, migration and EMT through another one of its transcriptional suppressors, known as SNAI2 [136]. When expressed in ovarian cancer, *miR-506* was associated with a favorable prognosis [136].

Studies on the presence of miRNAs in various malignancies have shown that by inhibiting the expression of proteins like HMGA2, RAS, c-Myc, and cdk6, the let-7 family had a suppressing effect on tumor growth and cell invasion [137,138]. One of its constituents, let-7b, was proved to inhibit tumor growth, both in vitro and in vivo, while low levels of let-7f were detected in those ovarian cancer cells that had metastatic abilities and high invasion capabilities, further endorsing the supposition that it might play a suppressing role in tumor invasion and metastasis [134,138]. Also inhibiting metastasis, *miR-183* and *miR-22* were highly expressed in low metastatic ovarian cancer [139]. Further research is needed in order to develop reliable diagnostic and prognostic tools that would ensure better chances of survival for ovarian cancer patients.

### 4.3. miRNAs in Cervical Cancer

Cervical cancer is the fourth most common malignancy diagnosed in women, with human papilloma virus (HPV) being present in 99% of all cases [66,145]. Although the mechanisms are not yet fully understood, there seems to be a link between the development of cervical cancer and the altered expression of miRNAs [145,156]. It is well known that beside circulating freely or bound to proteins in the blood, miRNAs can also be found in exosomes, small extracellular vesicles that originate from all types of cells, including malignant ones [145]. They carry the genetic material of the cell that released them, their components, like mRNAs being associated with malignancy in various studies [145]. Exosomes could be used as vehicles for tumor-suppressing miRNAs which would improve the survival rates and the prognostic of cervical cancer patients [157].

Studies on cervical cancer have observed the upregulation of a number of circulating miRNAs, like *miRNA-20a*, *miRNA-21*, *miRNA-203*, *miRNA-205*, *miRNA-485-5* as well as the upregulation of some tissue-specific miRNAs, among them being *miR-7*, *miR-10a*, *miR135b* and *miR-149* [145]. *miR-21a* is known to stimulate inflammation and proliferation and inhibit apoptosis through a variety of mechanisms like angiogenesis, cell invasion, and transduction of NF-kB pathway [66]. After lowering its expression in SiHa cells, an inhibition of tumor growth was noted [158].

Another miRNA that was detected in high quantities in cervical cancer is *miR-944*. Although many studies stated that it functions as an oncogene in various malignancies, among them being cervical cancer, by stimulating cell migration, invasion and proliferation [140,141], there are some that described tumor suppressor properties in cancers located in the colon, stomach, and breast [159,160,161]. The hypothesis that it might have oncogenic functions was recently studied using five types of cell lines and 116 cervical tissues [142]. *miR-944* was significantly higher expressed in cervical cancer tissues compared to controls, as well as in HPV-positive samples compared to those that were HPV-negative [142]. Its expression was associated with advanced FIGO stage, bulky tumor size, lymph node metastasis, and lower survival rates [142]. Considering all of the above, *miR-944* could potentially serve as a prognostic biomarker [142].

In contrast, other miRNAs like *miR-138* [143], *miR-148b* [162], *miR-195* [163], *miR-214* [164] were downregulated in cervical cancer, suggesting their role as tumor suppressors. As stated before, *miR-138* is lowly expressed in this malignancy and acts like a tumor suppressor by stimulating apoptosis and preventing cell migration and tumor growth [143,144,145]. It is negatively associated with lymph node metastasis and advanced FIGO stage, having the potential of being used as a prognostic biomarker [143,144,145]. *miR-34a* and *miR-206* could also be possible prognostic instruments as their low expression in cervical cancer was linked to late stage disease, lymph node dissemination, and low survival rates [165].

A major problem of cancer therapies is the development of chemotherapy resistance [166]. As already stated, miRNAs expression levels seem to influence the drug-sensitivity/resistance of the tumor cell [167]. Some of the miRNAs found to modulate the cells’ response to chemotherapy are *miR-34a* [168], *miR-375* [169] and *miR-664* [170]. For instance, a study on paclitaxel treated patients found that the expression levels of *miR-375* increased in a dose-dependent pattern, leading to a decreased sensitivity to the drug both in vitro and in vivo [169,171]. In contrast, an increased expression of *miR-664* in HeLa cells led to a higher sensitivity of cervical cancer cells to cisplatin and lowered cell migration [170].

### 4.4. miRNAs in Distinguishing Cancer Subtypes

Depending on the type of cells from which tumors originate and their underlying pathological mechanisms, cancers can be separated into distinct subtypes that possess different clinical and genetic features. Prompt and accurate diagnosis is virtually critical in order to initiate an adequate and effective treatment. Since miRNAs are known to be implicated in apoptosis regulation as well as initiation and progression of carcinogenesis [118,172], it stands to reason that these small RNAs be investigated with the aim of achieving a more precise diagnosis.

To illustrate this fact, we propose the typical example of lung cancer, where miRNA expression patterns differ not only between lung cancer patients and healthy people, but also between its two main categories, non-small-cell lung carcinoma (NSCLC) and small-cell lung carcinoma (SCLC), as well as their subtypes [173]. Up to date, in NSCLCs, an abundance of miRNAs has been identified, that can also vary depending on the stage of the disease [174,175]. A precise categorization into adenocarcinoma (ADC) and squamous cell carcinoma (SCC) is essential due to the differing treatment options [176]. For example, several studies highlighted a far higher expression of *miR-205* in SCCs than in ADCs [177,178], with some authors referring to it as a promising biomarker for the detection and recurrence prediction for lung cancer patients [179]. On the same note, searching for non-invasive and highly sensitive biomarkers for the early diagnosis of NSCLC, Jin et al. managed to show, through next generation sequencing, that ADCs exhibit the tumor-derived exosomal miRNAs *miR-30a-3p*, *miR-30e-3p*, *miR-181-5p* and miR-361-5p, while SCCs predominantly express *miR-10b-5p*, *miR-15b-5p* and *miR-320b* [180]. Along the same lines, studies have shown that precursors of certain miRNAs, such as *miRNA-944* and *miRNA-3662*, can differentiate early ADC from SCC, thus aiming to label pri-miRNAs as a novel class of lung cancer markers. In this regard, it was observed that pri-*miRNA-3662* was highly expressed in ADC patients in stages I and II, while the expression of pri-*miRNA-944* was higher in SCC patients in stages I and II [181]. On the other hand, in SCLC, Nishikawa et al. showed that *miR-375* was significantly increased in SCLC cell lines as opposed to those of different histologic types when they examined the miRNA expression profiles on lung cell lines [182]. Moreover, a recent cohort study has identified 13 miRNAs able to accurately differentiate between SCLC and NSCLC patients, 3 of which, namely hsa-miR-331-5p, hsa-miR-451a and *hsa-miR-363-3p*, reaching sensitivity and specificity of 100% [183].

Breast cancer is another example of inter- and intratumor heterogeneity, with different cell populations observed not only among different individuals, but sometimes also within the same tumor [184,185]. The critical importance of biomarkers in guiding treatment for breast cancer has long been established [186,187] and earlier discussed. Because of the generally poorer prognosis of TNBC, research has been focused lately on the discovery of biomarkers of breast cancer subtypes. To this extent, Shin et al. noticed a decreased expression of *miR-16*, *miR-21* and *miR-199a-5p* along with an increase in the levels of *miR-92a-3p* and *miR-342-3p* in both blood and tissue samples collected from TNBC patients [188]. Meanwhile, in HER2+ tumors, it has repeatedly been shown that *miR-4728* is co-expressed with *HER2*, acting as an oncogene [189,190,191]. Moreover, Søkilde et al. have recently demonstrated the expression of two other small RNAs in HER2-enriched tumors, namely *miR-2115* and *miR-7158*, which are otherwise very lowly expressed in healthy tissues [192]. Furthermore, they also observed that the miRNA cluster *miR-99a*/*let-7c*/*miR-125b* was predominantly expressed in Luminal A breast cancer (hormone receptor-positive/HER2-negative/levels of the protein Ki-67), its low expression predicting poorer outcomes [193], thus ensuring the distinction between it and Luminal B type tumors (hormone receptor-positive/HER2-positive/HER2-negative with high levels of Ki-67).

On the other hand, there are studies showing that some miRNAs that are highly expressed in cancer patients can be rather non-specific. For instance, Ferracin et al. assessed the levels of nine different miRNAs in the serum and plasma of lung, breast, colorectal and melanoma patients, and found that *miR-21-5p* was the single miRNA that was persistently raised in all cancer patients [194].

## 5. miRNAs in Cardiovascular Disease

According to the World Health Organization, cardiovascular diseases (CVDs) remain the number 1 cause of death worldwide. The need for novel diagnostic and prognostic biomarkers that can aid both the prevention and the treatment of CVD is therefore pressing. This review will mainly focus on acute coronary syndromes since findings continue to accumulate showing that heart attack and coronary artery disease, in general, are the most common causes of CVD deaths in both male and female patients [195].

Acute coronary syndromes refer to the damage suffered by the myocardial tissue as a result of decreased blood flow through the coronary arteries, its gravity depending on the grade of the occlusion. Circulating biomarkers that are traditionally used, while indispensable in current medical practice, still leave room for improvement, since, to date, no ideal cardiac biomarker can be singled out [196]. Cardiac troponins, considered one of the most advantageous assays for the diagnosis of acute myocardial necrosis, can be spilled into the bloodstream in any situation that involves damage to the cardiac muscle, making it an organ-specific biomarker [197]. For instance, an increase in oxygen demand caused by tachycardia can also lead to decreased perfusion, thus elevating the cardiac troponins [198]. A similar increase can be witnessed in infiltrative cardiomyopathies such as cardiac sarcoidosis [199]. A broad number of non-cardiac events can also lead to troponin elevation, including chronic renal failure [200], cerebrovascular accidents [201] but also strenuous endurance training [202].

With these disadvantages in mind, and due to the specificity of new molecular and genetic indicators, their use becomes more and more appealing. As seen in Table 2, quite a few studies have highlighted the upregulation of the same four miRNAs, namely *miR-1*, *miR-133a*, *miR-208a/b*, and *miR-499*, shortly after myocardial infarction [59,203,204,205], which are classically referred to as the myomiR family [206].

*miR-1* is a muscle-specific miRNA that can be identified in both cardiac and skeletal muscles, its release in AMI suggesting necrotic death of cardiac myocytes as source [207]. Although on a relatively small sample, Ai et al. managed to highlight an obvious increase in *miR-1* levels in acute myocardial infarction (AMI) patients, which returned to basal levels following treatment [61]. Other groups have also reported elevated plasma *miR-1* levels in AMI patients [61,208], while Corsten et al. have pointed out that this elevation was rather more moderate compared to the ones detected in the case of *miR-208b* and *miR-499* [59].

*miR-208a* is encoded within the α-cardiac muscle myosin heavy chain genes, while *miR-208b* and *miR-499* are encoded within introns of the β-cardiac muscle myosin heavy chain genes and are, therefore, regarded as heart-specific miRNAs [209], with all three of them belonging to the *miR-208* family [210]. An analysis performed by Wang et al. found that *miR-208a* had the highest accuracy in diagnosing AMI, with levels significantly increasing as early as 1 h post-occlusion in 90% of investigated AMI patients and in 100% AMI patients within 4 h [208]. On the other hand, when Liu et al. compared samples from AMI patients analyzing plasma levels of *miR-1*, *-208* and *-499*, they discovered that the latter had the highest predictive value of the three, and a reliability above that of traditional cardiac biomarkers, TnT, and CKMB [205]. Their result was in accordance with that of Devaux et al., which included a much larger sample size [211].

As for *miR-133*, Zhou et al. have highlighted its significantly higher levels in AMI patients, showing that it has an approximately 4-fold increase that returns to normal levels after about 7 days [70]. This contradicts an earlier study that found no meaningful differences between AMI patients and normal individuals when it came to *miR-133* levels [212]. Furthermore, Eitel at al. managed to demonstrate that *miR-133a* plasma levels could be an indicator of the clinical prognosis of ST-elevation myocardial infarction. They showed that the higher the levels, the greater the risk of serious myocardial damage, severe reperfusion injury and reduced myocardial salvage [213].

**Table 2:** miRNA regulation in acute coronary syndromes.

miRNAs	Serum Levels	References
*miR-1*	↑	Ai et al. [61], Wang et al. [208], Corsten et al. [59], Liu et al. [205], Kuwabara et al. [214], Widera et al. [215]
*miR-133a*	↑	Zhou et al. [70], Eitel at al. [213], Kuwabara et al. [214], Widera et al. [215], Xiao et al. [216]
*miR-208a/b*	↑	Corsten et al. [59], Liu et al. [205], Widera et al. [215], Liu et al. [210], Zhang et al. [217], Liu et al. [218]
*miR-499*	↑	Corsten et al. [59], Liu et al. [205], Adachi et al. [219], Youssef et al. [220], Zhao et al. [221]

Despite encouraging results, miRNAs are not currently used in the clinical diagnosis of acute myocardial infarction, for the most part because of the higher costs than those implied by the now gold-standard cardiac troponins, but also because of lack of sufficient clinical trials and standardized protocols.

## 6. miRNAs in Sepsis

Sepsis is a severe condition that is characterized by an abnormal host response to pathogenic microorganisms consisting of an excessive inflammatory response and subsequent multiple organ failure [222]. Although epidemiological data indicate that sepsis-associated mortality rates seem to decline, its incidence is still on the rise and it is therefore currently regarded as a major healthcare burden [223].

Accumulating evidence suggests that aberrant miRNA expression is associated with both sepsis [224,225] and non-infective systemic inflammatory response syndrome (SIRS) [226]. Previous studies have shown alterations in numerous circulating miRNAs during sepsis. Over time, biomarkers have been developed and used in the evaluation of sepsis, such as interleukins (IL)-1, -2, -4, -6, -8, -12, complement components C3a and C5a, C-reactive protein (CRP), procalcitonin (PCT), etc. [227,228], but it’s been proven that their individual deficiencies, sometimes to the point of inaccuracy, can be overcome when using combinations of these markers [229]. In order to further identify the pathogen(s), blood cultures are the next step, associating important limitations since they are time-consuming and can identify microbes that are only grown under standard conditions [230]. Due to these circumstances, the need for faster, cheaper and more specific alternatives persists, and miRNAs have shown themselves to be a promising method.

For instance, Yao L et al. observed that *miR-25* levels dropped in sepsis patients and that lower levels were also associated with overall increased mortality. In doing that, they also highlighted the superiority of *miR-25* over traditional biomarkers like C reactive protein and procalcitonin, according to the receiver operating characteristic (ROC) curve analysis performed within their study [231]. These findings were later experimentally reproduced in vivo and in vitro by Yao Y et al., showing that *miR-25* was indeed downregulated in sepsis models consisting of cecal ligation and puncture in rodents and lipopolysaccharide-induced cardiomyocytes [232].

It is currently accepted that *miR-223* is a crucial player in regulating exaggerated immune responses through its targets which include, among others, Stathmin, STAT3, Granzyme B, IGFR1 and Artemin [233]. In a study performed by Wang et al. investigating 50 sepsis diagnosed patients, *miR-223* had lower plasma values compared to SIRS patients and healthy individuals [234].

*miR-155* is another important regulator of the inflammatory response, with recent studies highlighting its key role during bacterial infection, where it acts as a negative regulator of inflammation [235]. Bandyopadhyay et al. showed that the inducing of *miR-155* during Francisella tularensis infection prompted the translational repression of MyD88 and SHIP-1, thus inhibiting the secretion of endotoxin-stimulated TNFα and attenuating the innate inflammatory response [236].

*miR-150* is a molecule that is expressed almost exclusively in immune cells [237]. Its dysregulation in sepsis has been recognized by different authors [238,239]. However, other reports show that the differences between *miR-150* levels in patients with sepsis and patients with non-infective SIRS were minute, while also highlighting that it was rather relevant in predicting a poor outcome of critical conditions regardless of the presence of infection, thus recommending its use as a prognostic marker in critical patients [224,240].

The deregulation of many miRNAs depending on the causative pathogen has been documented over the years, and we illustrated some of the most significant and recent findings in Table 3.

Many authors have suggested the use of miRNAs as biomarkers for a more targeted diagnosis that bypasses the time-consuming microbiological confirmation of infection. Still, some issues remain, including the lack of a standardized protocol regarding specimen collection, handling, and analysis.

## 7. miRNAs in Nervous System Disorders

Due to its intricacy, the nervous system (NS) represents one of the most important biological systems and is comprised of different cell types and advanced synaptic communications [250]. Nervous system diseases (NSDs), as a class of nervous disorders, are one of the main causes of disability and mortality [251]. In the field of neurology, even though the exact functions of miRNAs remain uncertain, studies conducted so far underline their potential to reveal the evolution of various diseases, making these molecules attractive candidates as potential biomarkers both for diagnosis and prognosis.

miRNAs are also responsible for NS functionality, being implicated in translation, RNA metabolism, gene development and regulation [252]. Many NSDs such as Alzheimer’s disease (AD), epilepsy, Parkinson’s disease (PD), glioblastoma (GBM), multiple sclerosis (MS), and myasthenia gravis (MG) are caused in part by aberrant expressions and/or dysfunctions of miRNAs [251,253,254,255,256,257].

It is known that AD represents one of the main causes for dementia, even if its exact pathogenesis is still unclear and there is no treatment available. In the cortex of AD patients, regardless of stage, *miR-107* is poorly expressed [258]. Epilepsy, characterized by recurrent seizures that generate abnormal neuronal activity in the brain, is one of the most common NSDs globally [253,254]. In many cases of PD patients, a neurodegenerative disorder is characterized by motor and nonmotor symptoms, the overexpression of miRNAs *miR-103a-3p*, *miR-30b-5p* and *miR-29a-3p* can serve as potential biomarkers. Moreover, the expression levels of *miR-I*, *miR-22* and *miR-29* are used in order to distinguish healthy subjects from non-treated PD ones [259]. Also, miRNAs play important roles in the most lethal brain tumor (GBM) in cellular proliferation, apoptosis, invasion, angiogenesis and stemness [255]. In MS and MG, evidence indicates the implication of *miR-132*, *miR-124*, and *miR-155* [257,260]. A brief summary of miRNAs implicated in neurologic disorders is provided in Table 4.

In Alzheimer’s disease, miRNA alterations can be detected both in the brain and cerebrospinal fluid (CSF) of the patients, and also in the biological fluids, such as plasma and serum, making them potential candidates as biomarkers [275]. The biomarkers currently in use are represented by tau (tubulin-associated unit) proteins and Aβ peptides [276]. However, they present certain drawbacks in the clinical practice, such as a lack of standardization of the technique in biological fluids and not enough evidence for cut-off values [277]. Also, in patients with mild cognitive impairment (MCI), their predictive values are still to be determined [278]. miRNAs on the other hand display higher sensitivity levels in comparison to protein biomarkers, mainly due to PCR amplification [279]. In addition, their analysis represents a relatively simple method, it is non-invasive and has a far lower cost than the currently used diagnostic techniques, such as MRI or molecular neuroimaging with PET.

Parkinson’s disease is a complex neurological ailment, which leads to peripheral deficits and cognitive impairment. At the moment, its diagnosis is established on motor functions rating and other clinical signs, which can only be assessed when the loss of the DAergic neuron is approximately 70% [280]. In this case, miRNAs have the potential to become reliable biomarkers for an early and precise diagnosis. Their alternative is represented by the detection of certain proteins, such as Lewy body formation or DJ-1 (that reveals the dysfunction of mitochondria) and α-SYN (for the aggregation of proteins) in brain tissue or CSF [281]. However, these are invasive procedures, or they are possible only after the patient has deceased. A large number of circulating miRNAs has been discovered in the past years for Parkinson’s disease, some of which could lead to an easier, non-invasive diagnosis for this pathology [282].

Similar impediments have been encountered in the case of multiple sclerosis. The currently used biomarkers include IgG and IgM antibodies, glycoproteins, markers of inflammation and chemokines [283]. However, the progression of this disease is very unpredictable and numerous phenotypes have been described, both of these issues leading to a lack of correlation with the present biomarkers. New and improved molecules are needed to solve this and provide a more specific diagnosis for the various phenotypes, determine the course of the disease and create a link with the level of disability [284,285]. In addition, less invasive methods could prove themselves more useful in clinical practice, regarding that the current source of biomarkers is CSF. miRNAs have the potential to meet all the discussed criteria.

Myasthenia gravis is an autoimmune condition characterized by the attack of autoantibodies upon the antigens found in the neuromuscular junction (AChR or MuSK). These antibodies represent a source of biomarkers for this disease, but even though they have the potential to diagnose it, the correlation between their titer and the progression of the disease or the patient’s response to therapy is still unreliable [286]. Further on, electromyography is another tool used for aiding the diagnosis in the clinical practice, but its practicality is limited by the low availability of expertise. The objective signs of this pathology rapidly fluctuate and can also be influenced by medication. The use of miRNAs as biomarkers for this condition could lead to an easier and improved method for diagnosis, a suitable solution for tracking the course of the disease and also a way to measure the response to treatment.

In 2007, Sayed et al. illustrated the importance of miRNAs in the NS by the finding that 70% of known miRNAs are expressed in the brain [287]. It is known that *miR-9* and *miR-134*, both brain-specific, were extensively studied in neurogenesis and their aberrant expression was associated with abnormal brain development and many neurodevelopmental diseases [288,289,290]. It is important to evaluate miRNAs expression in NSDs in order to get relevant molecular information and help clinical management of the disease processes [1].

## 8. Limitations of Circulating miRNAs as Biomarkers

Nowadays, circulating miRNAs can be obtained from venous plasma or serum, and it was demonstrated that miRNAs profiles in venous and arterial plasma were very similar, with no significant differences in theirs profiles between venous and arterial blood. In cancer detection, the use of venous miRNAs is still challenged in some cases. For example, in a study on plasma samples from male rats performed in 2017, the authors identified ten arterial and fourteen venous highly expressed miRNAs, and the miRNA profiles in arterial plasma showed higher correlation with that in tissue [291]. In humans, sample studies showed similar observations, leading to the conclusion that the blood sampling method should be carefully chosen for specific miRNA biomarkers [292,293].

miRNAs can be detected using various specific and sensitive approaches, including Northern blot analysis [294,295], in situ hybridization [296], real-time PCR [95,297], miRNA microarray [298,299] and next-generation sequencing (NGS) [300], and some of them are already used as diagnostic or prognostic markers, this demonstrating their utility in both clinical and personalized medicine. Some previous research regarding circulating miRNAs did not result in highly specific and validated disease markers due to the lack of taking into consideration important factors such as prior treatments, age, sex. Also, data reproducibility may pose an important problem, due to a lack of consistency [301]. In order to determine the accurate level of expression of miRNA in a specific tissue, body fluid or cell type and to ensure that both the positive and negative results are reliable, it is important to minimize experimental or technical variations by controlling the pre-processing of miRNA detection and normalization experiments, data processing and optimization [302], due to the minor differences in miRNAs expression levels between healthy people and patients [173]. Binderup et al. have emphasized the importance of the chosen normalization strategy in RT-qPCR data analyses [303]. The most frequent normalization technique involves the use of an exogenous spike-in of a synthetic miRNA such as *cel-miR-39* or an endogenous one like *miR-16* as reference genes during the initial phase of RNA extraction [304]. However, it is generally strongly implied that the use of a single reference gene is insufficient for accurate miRNA results, with researchers preferring strategies that apply a combination of control miRNAs, both exogenous and endogenous [305].

One issue for using miRNAs as diagnostic markers is the relatively poor diagnostic specificity and reproducibility of some identified miRNAs. The methods used for miRNA detection need to be standardized prior to use for diagnostic purposes in order to generate and discover useful research hypotheses and new biomarkers for in vitro diagnostics [1]. The main challenges in using miRNAs in in vitro diagnostics are discovering specific miRNAs that can be used as biomarkers in a broad range of patients and developing accurate, simple and inexpensive methods that involve standardized pre- and post-analytical procedures.

miRNA expression is dysregulated in patients’ brains compared to healthy ones, and there is genetic evidence showing that these mutations that occur and disrupt miRNAs transcription are found in NDs. Manipulation of miRNAs could change the phenotypes in some NDs in experiments performed using animal models, so it is reasonable to expect to use miRNAs as therapeutic agents due to the small size of miRNA molecules [306]. Although using miRNAs as biomarkers and in therapy is very promising, there are still a lot of challenges, including the possibility of off-target effects which may cause severe unanticipated responses. Moreover, one miRNA can function differently according to the stage of the disease, making it difficult to be used as a precise target [307].

To avoid limitations, it is important to take into consideration aspects such as the sample size (if it is not powerful enough, it would not be able to detect variants with minor effects), prospective studies, which are important in confirming the observational study results, and the validation process, which has to be performed only after warranty of the epidemiological and functional studies [308].

## 9. Why Are miRNAs Not Used in Current Practice?

For clinical application, the most important evaluation criteria for circulating miRNAs as diagnostic and prognostic biomarkers are high sensitivity and specificity, to avoid false positive or negative diagnosis. In clinic, an appropriate biomarker for a specific cancer type should be both significantly differentially expressed and in correlation with the outcome of patients. The low specificity of single miRNA molecules compared with the circulating miRNA molecules was highlighted. Many cell-free miRNAs showed altered expression patterns in different types of cancers instead of a certain type, and also similar expression between benign and malignant tumors; *miR-21-5p*, *miR-155-5p* and *miR-210-3p* for example, are all involved in many cancer types, but for *miR-21-5p*, significantly increased levels in plasma of CRC patients were observed, but it could not distinguish between malignant cancer and benign polyps [309]. In patients with NSCLC [175,310,311,312,313], liver cancer [314] or gastric cancer [315,316], it was reportedly upregulated, but the same molecule was downregulated in patients suffering from breast cancer [188,313]. This means that the contribution of miRNAs in various types of cancers differs.

Therefore, it is essential to have a larger sample size to be able to decide between the healthy or diseased status. Due to many criteria used in clinical applications like age, gender, ethnicity, lifestyle, pre-treatment, history of diseases and so on it is mandatory to avoid the obstacle of limited sample size. Regarding detection results, they can be affected by measurement principle, method used, instrument, technicians [173].

## 10. Perspectives and Conclusions

Since their discovery, a large number of miRNAs has been described. Although progress has been made towards figuring out the role of miRNAs in pathological cases, many conditions remain insufficiently characterized since it is still a relatively young field. However, great potential lies in identifying these disease-specific molecules and integrating their role in the strategy of diagnosis, prognosis, and treatment.

Numerous miRNAs are frequently altered in various conditions, starting from the world’s main cause of morbidity and mortality, namely cardiovascular disease, to neurological disorders, sepsis and last but not least, to cancer. In order to rapidly diagnose the diseases associated with an increased death rate, new and improved methods are necessary. miRNAs have the required properties to constitute such a method.

Although many miRNAs can be found intracellularly, they have also been observed on the outside of the cell, later being coined circulating miRNAs, and demonstrated high stability and specificity. The high stability of circulating miRNA is mainly due to the encapsulation in lipid vesicles or the formation of complexes with different kinds of proteins that protect them against denaturation. Therefore, a wide variety of miRNAs are detected in exosomes, microvesicles, or high-density lipoprotein particles from a diversity of biological fluids, such as blood, urine, saliva, peritoneal fluid, amniotic fluid, bronchial lavage, CSF, and tears [2,317,318].

Several studies have shown that miRNAs are released in the bloodstream from different organs such as brain, heart, endothelial cells, ovary, uterus, and mammary glands, in both healthy subjects and patients. In cancer patients, studies demonstrated that circulating miRNAs seem to originate from tumor tissues and may evaluate tumor progression. Moreover, after resection of the tumor, oncogenic miRNAs levels tend to decrease [319].

Since it was discovered that miRNA could be detected in both extra- and intracellular environments, their potential use as biomarkers became one of the main focuses of current research. Although many studies have been conducted, there is still a lack of consistency between many miRNA signatures. These differences may be due to the source of miRNA (blood, plasma, CSF,) and the size of the cohorts of patients with various comorbidities that may influence these molecules. For instance, some studies of Alzheimer’s disease analyze miRNAs originating from distinct blood compartments [320,321] while others focus on miRNAs from CSF [262,322,323]. It has been shown that *hsa-miR-146a* was upregulated in one of the latter studies [262], while in the second one it was downregulated [322] and the third showed no modification [323]. Not to mention that the sampling method, as well as the preservation and the processing of the samples, can also lead to discrepancies regarding the results.

Nowadays, various methods have been developed depending on the purpose of the project: hybridization-based approaches (microarray, nCounter Nanostring technology), reverse transcription quantitative PCR arrays (RT-qPCR), NGS. An initial inexpensive screening can be carried out through microarray based on RNA-DNA hybrid capture, while NGS can be used for detecting new miRNAs and different isoforms [324,325,326]. Due to the many qualities of RT-qPCR, which include high sensitivity, specificity, accessibility, and reproducibility, RT-qPCR is currently the most used technique, remaining the gold standard method for the verification of microarray and NGS results.

There is an imperious need for faster ways to detect different pathologies, especially seeing that many types of cancer are discovered in late stages. miRNAs as biomarkers can fulfil this desideratum despite present limitations, thus being an impressive research field. As current techniques evolve, we anticipate that miRNAs will become a routine approach in the development of personalized patient profiles, therefore allowing targeted therapeutic interventions.

## Figures and Tables

**Table 1:** MicroRNA (miRNA) regulation in cancer.

miRNA	Disease	Regulation	Main Characteristics	Reference
miR-10b	Breast Cancer	Upregulated	Overexpressed only in metastatic disease In non-metastatic disease—cell invasion and metastasis inductor High levels correlated to disease progression and poor outcome	[124,125,126]
miR-196a	Breast Cancer	Upregulated	Oncogenic role Alters apoptosis and angiogenesis High levels associated with advanced disease	[127,128,129,130]
miR-4417	Breast Cancer	Downregulated	Prognostic tool for TNBC Levels correlated to the evolution of the disease	[52,131]
miR-200 cluster	Ovarian Cancer	Upregulated	Low levels associated with cell invasion and metastasis Low levels predict poor outcomes and disease recurrence Angiogenesis inhibitors	[132,133,134,135]
miR-506	Ovarian Cancer	Dysregulated	Cell invasion, migration and EMT inhibitor High levels confer a good prognosis	[136]
let-7 cluster	Ovarian Cancer	Dysregulated	Suppressors of tumor growth and cell invasion	[134,137,138]
miR-183	Ovarian Cancer	Dysregulated	Overexpressed in low metastatic ovarian cancer	[139]
miR-22	Ovarian Cancer	Dysregulated	Overexpressed in low metastatic ovarian cancer	[139]
miR-21a	Cervical Cancer	Upregulated	Inflammation and proliferation stimulator Apoptosis inhibitor	[66]
miR-944	Cervical Cancer	Upregulated	Levels associated with advanced FIGO stage, bulky tumor size, lymph node metastasis Poor prognostic factor Higher levels in HPV-positive cervical cancer	[140,141,142]
miR-138	Cervical Cancer	Downregulated	Tumor suppressor role Apoptosis stimulator Negatively associated with lymph node metastasis and advanced FIGO stage	[143,144,145]

**Table 3:** miRNA regulation in bacterial infections.

Pathogen	miRNAs	Regulation	Reference
*Staphylococcus aureus*	*miR-133a*, *miR-133b*, *miR-122*, *miR-205*, *miR-1899*, *miR-714*, *miR-291b*	↑	Wu and colleagues [241]
*Streptococcus pneumoniae*	*hsa-miR-199b-5p*, *hsa-miR-30a-5p*	↑	Poore et al. [242]
	*hsa-miR-942-5p*, *hsa-miR-342-5p*, *hsa-miR-503-5p*	↓	
*Escherichia coli*	*miR-16*, *miR-17*, *miR-20a*, *miR-26a*, *miR-26b*, *miR-106a*, *miR-106b*, *miR-451*	↑	Wu and colleagues [241]
*Listeria monocytogenes*	*miR-133*, *miR-998*	↓	Mannala and colleagues [243]
	*miR-954*, *miR-3000*	↑	
*Mycobacterium tuberculosis*	*miR-7-1*, *let-7f-2*, *miR-126*, *miR-130b*, *miR-18a*, *miR-19b-1*, *miR-505*, *miR-545*, *miR-550*, *miR-760*, *miR-875-5p*	↓	Furci and colleagues [244]
	*miR-127-3p*, *miR-290b*, *miR-378*, *miR-337-5p*, *miR520c-3p*, *miR-573*, *miR-601*, *miR-645*	↑	
*Brucella melitensis*	*let-7b*, *miR-93*, *miR-151-3p*, *miR-92a*, *miR-142-5p*, *miR-99a*, *miR-181b*, *miR-1981*	↑	Zheng and colleagues [245]
*Pseudomonas aeruginosa*	*miR-302b*, *miR-301b*, *miR-762*, *miR-155*	↑	Zhou et al. [235], Mun et al. [246], Yang et al. [247]
*Plasmodium vivax*	*miR-451*, *miR-16*	↓	Chamnanchanunt and colleagues [248]
	*miR-7977*	↑	Kaur and colleagues [249]

**Table 4:** miRNA regulation in neurologic disorders.

Disease	miRNAs	Regulation	Reference
Alzheimer’s Disease	*miR-107*, *miR-298*, *miR-328*	↓	Wang et al. [258], Boissonneault et al. [261]
	*miR-9*, *miR-34a*, *miR-125b*, *miR-146a*, *miR-155*	↑	Alexandrov et al. [262]
Parkinson’s Disease	*miR-103a-3p*, *miR-30b-5p*, *miR-29a-3p*, *miR-4639-5p*	↑	Margis et al. [259], Chen et al. [263]
	*miR-29a-3p*, *miR-29c-3p*, *miR-19a-3p*, and *miR-19b-3p*	↓	Botta-Orfila et al. [264]
Glioblastoma	*miR-21*, *miR-221*, *miR-222*, *miR-335*	↑	Papagiannakopoulos et al. [265], Zhang et al. [266], Shu et al. [267]
	*miR-124*, *miR-137*, *miR-218*, *miR-451*	↓	Silber et al. [268], Xia et al. [269], Nan et al. [270]
Multiple Sclerosis	*miR-19a*, *miR-21*, *miR-22*, *miR-142-3p*, *miR-146a*, *miR-146b*, *miR-155*, *miR-210*, *miR-326*	↑	Ma et al. [271]
	*miR-15a*, *miR-15b*, *miR-181c*, *miR-328*	↓	Ma et al. [271]
Myasthenia Gravis	*miR-21-5p*, *miR-150-5p*, *miR-151a-3p*, *let-7a-5p*, *let-7f-5p*, *miR-423-5p*	↑	Punga et al. [272], Punga et al. [273]
	*miR-15b*, *miR-122*, *miR-140-3p*, *miR-185*, *miR-192*, *miR-20b*, *miR-885-5p*	↓	Nogales-Gadea et al. [274]
